# Supplementary material for: The genome sequence of the Gold Spot moth, Plusia festucae (Linnaeus, 1758)
Source: Wellcome Open Res. 2024 Dec 2;9:704. [Version 1] doi: 10.12688/wellcomeopenres.23409.1 (PMC11803378; doi:10.12688/wellcomeopenres.23409.1)
Supplement: Peer review history

# Reviewer report: Reviewer response for version 1

**DOI:** 10.21956/wellcomeopenres.25816.r114154

**Published:** 2024-12-19

**Authors:** Arun Arumugaperumal

**Affiliations:** 1 Department of Biotechnology, Rajalakshmi Engineering College, Tamilnadu, India

This is the first report of the whole genome sequence of the moth *Plusia festucae*. The assembly presented is of length 422.5 Mb. The authors have also assembled the mitochondrial genome and predicted 19,273 genes. The genome completeness analysis shows 98.8% complete.

 The authors say that the assembly was decontaminated using ASCC pipeline. Is the information on contaminating sequence available for other researchers?

 The data article can be accepted for indexing.

Are sufficient details of methods and materials provided to allow replication by others?

Yes

Is the rationale for creating the dataset(s) clearly described?

Yes

Are the datasets clearly presented in a useable and accessible format?

Yes

Are the protocols appropriate and is the work technically sound?

Yes

Reviewer Expertise:

Bioinformatics; Genomics

I confirm that I have read this submission and believe that I have an appropriate level of expertise to confirm that it is of an acceptable scientific standard.

# Reviewer report: Reviewer response for version 1

**DOI:** 10.21956/wellcomeopenres.25816.r114152

**Published:** 2024-12-18

**Authors:** Sivasankaran Kuppusamy

**Affiliations:** 1 Entomology Research Institute, Loyola College, Chennai, India

Authors have done the chromosome level genome assembly of *Plusia festucae *(Linnaeus, 1758). Through the comprehensive data they assembled 422.50 megabases and detected 31 chromosomes. The interesting thing is that the authors have observed the synteny (Z chromosome) through manual observation in this assembly.

**Minor comments on the manuscript**

 In the Background the first sentence can start as “The Gold Spot moth, *Plusia festucae* (Linnaeus, 1758) (Figure 1)………

 In the second paragraph first sentence The *Plusia festucae *genome can be deleted. The sentence starts from “Here we present a …………………….

 The second sentence “It was sequenced….. can be modified as “The sequence was done as part of the Darwin Tree of Life Project….

 Throughout the text, the authors have used the full version of the genus name *Plusia festucae*. The genus name may be stated in its full form for the first time, and then in its abbreviated form, such as *P. festucae*.

 Above all, I confirm that the manuscript meets the necessary scientific standard and is suitable for indexing.

Are sufficient details of methods and materials provided to allow replication by others?

Yes

Is the rationale for creating the dataset(s) clearly described?

Yes

Are the datasets clearly presented in a useable and accessible format?

Yes

Are the protocols appropriate and is the work technically sound?

Yes

Reviewer Expertise:

Mitogenome sequencing and phylogenetic analysis of superfamily Noctuoidea moths

I confirm that I have read this submission and believe that I have an appropriate level of expertise to confirm that it is of an acceptable scientific standard.
